# Reflections and Prospects of the Gerontological Training and Education

**DOI:** 10.1093/geroni/igaa057.1798

**Published:** 2020-12-16

**Authors:** Kathy Lee, Tyrone Hamler, Cara Wallace

**Affiliations:** 1 University of Texas at Arlington, Arlington, Texas, United States; 2 Case Western Reserve University, Cleveland, Ohio, United States; 3 Saint Louis University, St. Louis, Missouri, United States

## Abstract

The demand for professional training, mentorship, and research in the field of aging is expected to increase remarkably. Recent statistics indicate less than 8% of social work students nationwide specialize in gerontology; however, a significant amount of social work graduates, regardless of their specialization at school, serve older adults in various social and health care settings. As a panel, former fellows present experiences as participants of the AGESW Pre-Dissertation Fellows Program. The program helps students comprehend basic principles of doctoral education and develop strong professional networks with other gerontology-focused colleagues and mentors across the country. Doctoral students are also trained to be competent working with older adults through research, teaching, and professional development. Many fellows move into faculty positions and their accomplishments are varied and impressive. It is difficult to separate these from the education, connections, mentorship, and support received from the AGESW network and through participation in this program.

